# Evaluation of Patella Anatomy for Total Knee Arthroplasty Approaches

**DOI:** 10.7759/cureus.59852

**Published:** 2024-05-07

**Authors:** Barış Kafa, Hasan B Ilgaz, Mehmet Ülkir, Shanzeda Khan Efil

**Affiliations:** 1 Orthopaedics and Traumatology, Gulhane Training and Research Hospital, Ankara, TUR; 2 Anatomy, Hacettepe University, Ankara, TUR; 3 Anatomy, Bilkent City Hospital, Ankara, TUR

**Keywords:** wiberg classification, morphometry, total knee replacement (tkr), anatomy, patella

## Abstract

Background: The patella, or kneecap, is a sesamoid bone situated deep to the fascia latae and the tendinous fibers of the rectus femoris. The medial and lateral facets of the patella articulate with the medial and lateral condyles of the femur, respectively, to form the patellofemoral component of the knee joint. When joint cartilage is destroyed due to osteoarthritis, inflammatory arthritis, post-traumatic degenerative joint disease, or osteonecrosis/joint collapse with cartilage loss, a surgical treatment called knee arthroplasty, or total knee arthroplasty (TKA), is used to rebuild the knee joint.

Objectives: The purpose of our study is to provide a detailed morphometric analysis of the human patella.

Methods: A total of 168 patellae (86 left, 82 right) were examined. Eleven parameters were determined to evaluate patella morphometry, and the bones were also evaluated with the Wiberg classification.

Result: Type I patella was observed in 13 samples (7.74%); 109 (64.88%) and 46 (27.38%) were Type II and Type III, respectively. In the statistical analysis, significant differences were found between the right and left patellae in terms of patellar thickness, vertical ridge length, and Wiberg angle (p<0.05). There were also significant differences between the Wiberg types and the medial articular width and lateral articular width (p<0.05).

Conclusion: In order to avoid potential difficulties during knee surgery, it is crucial to understand the typical morphological and morphometric properties of the patella. We believe that this study will be useful to surgeons who perform surgical approaches to the knee and to clinicians who evaluate the diseases of the region.

## Introduction

The patella, referred to as the kneecap, serves as a sesamoid bone located in a deep position relative to both the fascia latae and the tendinous fibers of the rectus femoris. Anatomically, it exhibits a flat, approximately triangular shape with a rounded apex situated on its anteroinferior margin. Its proximal aspect is termed the base, characterized by roughened surfaces facilitating the attachment of the quadriceps tendon. Additionally, its medial and lateral borders present roughened regions designed for the insertion points of the vastus medialis and vastus lateralis muscles, respectively [1].

The patella has distinct anterior and articular surfaces and three borders. The anterior surface has vertical ridges and no articular characteristics. In contrast, the articular surface has an oval-shaped articular region bisected by a smooth vertical ridge, splitting it into medial and lateral facets. Typically, the lateral facet is larger than the medial one [2]. The medial and lateral facets of the patella establish articulations with the corresponding medial and lateral condyles of the femur. This interaction constitutes the patellofemoral joint of the knee [1].

The patellar articular cartilage is the thickest in the body, which shows the magnitude of the stresses to which it is subjected [3]. In a patella unaffected by pathologies or erosion, the native thickness can be approximated as half its width [4]. During knee extension, the quadriceps muscles' actions are enhanced by the patella. It causes the quadriceps' moment arm to increase from 30% near extension to 15% at 30° flexion, particularly in the initial degrees of flexion [5].

Because of its essential function in the human body in terms of stability and mobility, it can degenerate due to many medical conditions [6]. When joint cartilage is destroyed due to osteoarthritis, inflammatory arthritis, post-traumatic degenerative joint disease, or osteonecrosis/joint collapse with cartilage loss, a surgical treatment called knee arthroplasty, or total knee arthroplasty (TKA), is used to rebuild the knee joint. When conservative therapies have failed, it is a dependable operation with predictable results that are frequently used to treat symptomatic osteoarthritis affecting numerous compartments of the knee [4,7].

The purpose of our study, thereby, is to provide a detailed morphometric analysis of the human patella in our bone sample, which will not only help facilitate the understanding of the pathogenesis of disorders of the knee but also help physicians in the fields of orthopedics, plastic surgery, forensic medicine, and anatomy.

## Materials and methods

A total of 168 dried patellae (86 left, 82 right) were examined from a sample of unknown sex and age in the collections of the Faculty of Medicine, Department of Anatomy, Hacettepe University, Ankara, Turkey. There were no obvious signs of physical damage or pathological trauma in any of the specimens. 

In line with previous studies on this subject, 11 parameters were determined to evaluate patella morphometry, and the bones were also evaluated with the Wiberg classification. In this classification, Type I is characterized by equal sizes of the lateral and medial facets. In Type II, the medial facet is smaller than the lateral one, and there's a slight medial displacement of the ridge, positioning it closer to the patella's medial border. In Type III patella, the ridge is displaced significantly towards the medial side, resulting in the absence of space for the medial facet [8].

**Figure 1:**
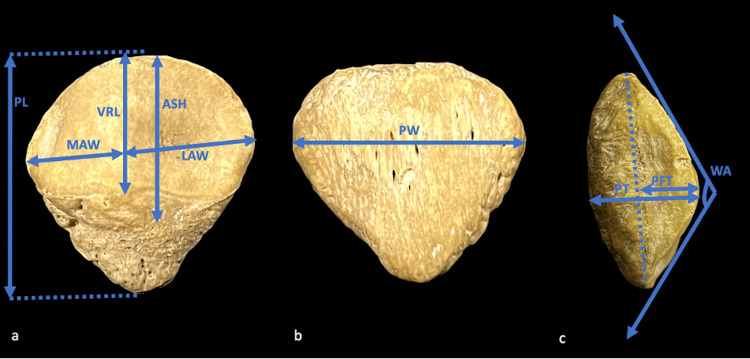
Demonstration of measured parameters: articular surface (a), anterior surface (b), lateral view (c) of the patella PL: patellar length; PW: patellar width; PT: patellar thickness; MAW: medial articular width; LAW: lateral articular width; ASH: articular surface height; VRL: vertical ridge length; WA: Wiberg angle; PFT: patellar facet thickness.

In addition to the classification types, 11 other parameters were measured, as outlined in Figure 1. These parameters included patellar length (PL), patellar width (PW), patellar thickness (PT), medial articular width (MAW), lateral articular width (LAW), articular surface height (ASH), vertical ridge length (VRL), Wiberg angle (WA), patellar facet thickness (PFT), patellar relative thickness (PRT = PT/PW), and patellar facet thickness ratio (PFTR = PFT/PT) [5, 9].

A sliding digital caliper with an accuracy of 0.01 mm was used for linear measurements. The WA was measured with a goniometer. All the measurements were expressed as mean ± standard deviation and in mm or degree.

Ethics committee approval was obtained from the Hacettepe University non-interventional clinical research ethics committee with the decision number 2024/04-09.

Statistical analysis

The statistical analysis was conducted using IBM SPSS Statistics Software for Windows, version 23.0 (IBM Corp., Armonk, NY). The distribution of the data was calculated with the Kolmogorov-Smirnov and Shapiro-Wilk normality tests, then the Student-t test or Mann-Whitney, and one-way ANOVA or Kruskal-Wallis tests were used for comparison of the groups. The correlation of the data was evaluated with Pearson's or Spearman's coefficient analysis.

## Results

**Figure 2:**
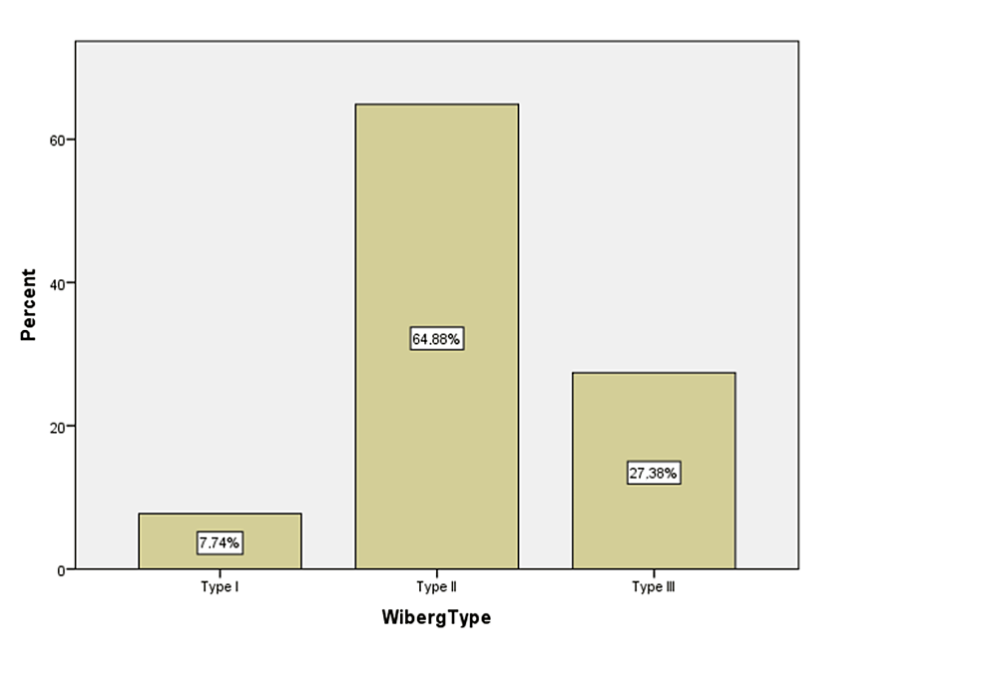
Distribution of patellae according to Wiberg types Type I: The medial and lateral facets are equal in size; Type II: The ridge is situated slightly towards the medial border of the patella, and the medial facet is smaller than the lateral; Type III: The ridge is displaced medially to such a degree that there is no space left for the medial facet.

Patellas were morphometrically classified using the Wiberg classification, as shown in Figure 2. The differences and distributions of the 168 patellae's articular surfaces morphometric analysis are presented. Type I was observed in 13 samples (7.74%), while Types II and III accounted for 109 (64.88%) and 46 (27.38%) samples, respectively. Figure 2 illustrates that the most prevalent type observed was Type II.

The mean values of the morphometric measurements of the patellae and the comparison of the means according to their sides are shown in Table 1. The mean values of the morphological findings related to the size of the patellae (PL, PW, PT, PFT, PRT, and PFTR) were 38.83±3.47 mm, 40.22±3.92 mm, 19.41±2.13 mm, 10.89±1.33 mm, 2.10±0.15 mm, and 0.56±0.06 mm, respectively. Table 1 shows that significant differences were found between the right and left patellae in terms of the PT, VRL, and WA (p<0.05). 

**Table 1:** Morphometric measurements of the patellae R: right; L: left; T: total; PL: patellar length; PW: patellar width; PT: patellar thickness; MAW: medial articular width; LAW: lateral articular width; ASH: articular surface height; VRL: vertical ridge length; WA: Wiberg angle; PFT: patellar facet thickness; PRT: patellar relative thickness; PFTR: patellar facet thickness ratio; SD: standard deviation; * indicates p<0.05.

Parameter	Side	Mean±SD	Minimum	Maximum	p-value
PL (mm)	R	39.16±3.47	32.41	49.07	.240
	L	38.53±3.46			
	T	38.83±3.47			
PW (mm)	R	40.16±4.22	23.90	50.92	.886
	L	40.28±3.64			
	T	40.22±3.92			
PT (mm)	R	19.78±2.35	13.59	30.25	.029*
	L	19.06±1.84			
	T	19.41±2.13			
MAW (mm)	R	20.33±2.36	11.80	29.36	.312
	L	20.05±2.35			
	T	20.19±2.35			
LAW (mm)	R	25.79±2.47	20.20	54.41	.121
	L	25.52±4.02			
	T	25.65±3.35			
ASH (mm)	R	28.69±2.44	21.58	78.18	.412
	L	28.95±6.05			
	T	28.83±4.64			
VRL (mm)	R	25.87±3.40	5.91	34.14	.033*
	L	24.71±3.57			
	T	25.28±3.53			
WA (degree)	R	120.76±5.74	108.20	142.60	.048*
	L	122.66±6.61			
	T	121.73±6.26			
PFT (mm)	R	11.02±1.56	8.53	14.13	.503
	L	10.75±0.99			
	T	10.89±1.33			
PRT (mm)	R	2.07±0.14	1.78	2.41	.193
	L	2.15±0.16			
	T	2.10±0.15			
PFTR	R	0.55±0.06	0.44	0.68	.094
	L	0.57±0.06			
	T	0.56±0.06			

**Figure 3:**
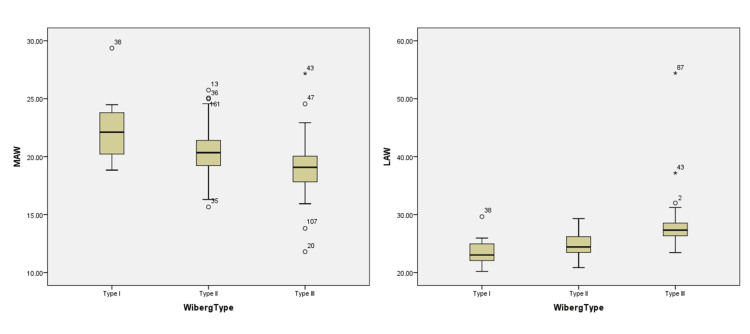
Distribution of the MAW and LAW according to Wiberg types MAW: medial articular width; LAW: lateral articular width

Figure 3 indicates the analyses between the Wiberg types and the MAW and LAW. As the number of Wiberg classification types increased, MAW also exhibited an increase in size. Conversely, the relationship was reversed for the LAW. When the parameters were evaluated as their Wiberg types, there were statistically significant differences between the Wiberg types and the MAW and LAW (p<0.05). 

## Discussion

The patella, also known as the kneecap, is a flat, triangular-shaped bone located deep within the thigh muscles. It has rough surfaces for muscle attachments, including the rectus femoris, vastus intermedius, vastus medialis, and vastus lateralis [1]. It has distinct anterior and articular surfaces and three borders. Its articular cartilage is the body's thickest, highlighting the significant stresses it endures [3]. During knee extension, the patella increases the arm movement by increasing the movement of the quadriceps muscles [5].

Studies conducted on various populations, including Asian, Japanese, Indian, and South African, by researchers such as Kim et al., Uehara et al., Vaidya et al., Taj et al., and Olateju et al. have revealed variations in the patella [9-13]. In previous studies, the measurements were as follows: PL ranged from a minimum of 35.7 mm [14] to a maximum of 46.1±4.7 mm [15]; PW ranged from a minimum of 38.53 mm [2] to a maximum of 48.95±0.72 mm [16]; PT ranged from a minimum of 18.40±0.17 mm [16] to a maximum of 26.6±1.5 mm [15]; MAW ranged from a minimum of 17.28±1.91 mm [17] to a maximum of 26.00±0.16 mm [16]; LAW ranged from a minimum of 20.03±3.13 mm [17] to a maximum of 27.7±1.8 mm [15]; and ASH ranged from a minimum of 25.87±4.02 mm [18] to a maximum of 31.26±3.17 mm [5]. In our study, these parameters were measured in accordance with the literature. Our study gains importance when compared to previous studies due to the comprehensive morphometric analysis of a relatively large sample size of 168 patellar. The VRL was measured at 27.7±2.3 mm on the right side and 27.5±3.5 mm on the left side by Taj et al., which was more than our measurement of 25.87±3.40 mm on the right side and 24.71±3.57 mm on the left side [9]. A comparison of the morphometric properties of the patella is summarized in Table 2.

**Table 2:** Comparison of morphometric parameters of the patella N: sample size; R: right; L: left; T: total; PL: patellar length; PW: patellar width; PT: patellar thickness; MAW: medial articular width; LAW: lateral articular width; ASH: articular surface height; VRL: vertical ridge length

Study (year)	Country	N	Side	PL (mm)	PW (mm)	PT (mm)	MAW (mm)	LAW (mm)	ASH (mm)	VRL (mm)
Baldwin and House (2005) [14]	USA	92	T	35.7	46.1	22.6	20.5	27.5	-	-
Olateju et al. (2013) [13]	South Africa	46	T	43.73±3.65	45.14±3.96	R:23.85±2.18 L:24.10±2.06	20.38±3.36	26.02±2.68	-	-
Shang et al. (2014) [19]	China	80 (40 R, 40 L)	R	39.98±3.55	44.12±3.98	22.65±1.83	18.92±2.20	25.21±2.88	26.53±4.09	-
			L	39.90±3.85	44.15±3.99	22.79±1.82	19.15±2.24	25.06±2.68	26.21±3.43	-
Chhaparwal et al. (2018) [2]	India	50 (25 R, 25 L)	R	36.61	38.80	19.21	20.94	22.73	-	-
			L	36.72	38.53	19.31	20.44	23.97	-	-
Agarwal et al. (2018) [17]	India	60 (34R, 26 L)	R	40.51±2.96	42.04±2.69	20.10±1.48	17.28±1.91	21.43±2.22	-	-
			L	38.24±7.68	40.26±4.51	19.49±2.05	17.78±1.86	20.03±3.13	-	-
Katchy et al. (2020) [15]	Nigeria	60 (30 R, 30 L)	T	46.1±4.7	46.9±2.9	26.6±1.5	25.5±1.8	27.7±1.8	-	-
Joshi and Vaniya (2021) [16]	India	90 (45 R, 45 L)	R	38.37±0.55	48.95±0.72	18.68±0.17	22.57±0.22	27.00±0.19	-	-
			L	37.40±0.54	47.40±0.48	18.40±0.17	26.00±0.16	27.00±0.17	-	-
Maia et al. (2021) [20]	Brazil	59 (28 R, 31 L)	T	-	42.64	-	18.47±0.36	24.17±0.31	-	-
Bisht et al. (2022) [5]	India	100 (50 R, 50 L)	R	40.21±2.93	42.83±3.02	20.31±1.49	22.38±1.77	25.87±2.32	31.26±3.17	-
			L	40.02±2.68	41.65±2.78	20.14±1.51	21.88±1.52	25.78±1.94	30.69±1.65	-
Taj et al. (2022) [9]	India	50 (26 R, 24 L)	R	40.0±3.4	41.1±3.7	20.4±1.6	21.5±2.1	24.3±1.9	-	27.7±2.3
			L	41.3±4.9	41.3±2.8	20.1±1.8	20.5±2.6	24.8±2.0	-	27.5±3.5
Biswas et al. (2023) [18]	India	200 (100 R, 100 L)	R	39.11±3.77	39.64±2.95	19.48±1.88	20.58±2.05	24.20±2.09	25.94±3.63	-
			L	39.08±3.49	40.14±3.85	18.98±1.93	19.98±2.17	23.68±2.51	25.87±4.02	-
This study	Turkey	168 (82 R, 86 L)	R	39.16±3.47	40.16±4.22	19.78±2.35	20.33±2.36	25.79±2.47	28.69±2.44	25.87±3.40
			L	38.53±3.46	40.28±3.64	19.06±1.84	20.05±2.35	25.52±4.02	28.95±6.05	24.71±3.57

Wiberg used comparisons between the medial and lateral facets of the articular surface of the patella to develop a categorization scheme for the various patellar facet sizes. The concave medial and lateral facets of Type I, which is 10% prevalent, are almost identical in size. Wiberg Type II patellas have a smaller medial facet than the lateral facet, which is flat or slightly convex. At 65%, this morphology is the most common. A Type III patella, which accounts for 25% of all occurrences, is convex in shape as opposed to Type II and also has a smaller medial facet than the lateral aspect [8]. The majority of patella was Type II in previous studies [5,8,9,17,20,21]. In the study by Agarwal et al., Type I patella was not detected, and no Type III patella was detected in the studies by Murugan et al. and Maia et al. [17,20,21]. The incidence of Type II patella (109, 64.88%) was found to be lower than in other studies in our study (Table 3). Unlike these studies, Fucentese et al. evaluated patellar morphology in the control group and the trochlear dysplasia group. They detected mostly Type I patella in the control group (20 of 22 patella) and Type II patella in the trochlear dysplasia group (12 of 22 patella). A significant difference was detected between these groups for patellar type [22]. According to studies by Fucentese et al. and Yılmaz et al., patients with trochlear dysplasia had shorter medial facet lengths and smaller transverse and vertical patellar sizes when compared to healthy knees [22,23]. In a study by Askenberger et al., individuals without patellar dislocation had a considerably lower incidence of Wiberg Type III patella than skeletally immature children with a primary patellar dislocation [24]. Additionally, Servien et al. detected that the patellae of individuals with patellar dislocation were more likely to have a patella with a hypoplasic medial border, a Wiberg type III patella, or a short patellar apex [25]. Pfirrmann et al. also demonstrated that in patients with trochlear dysplasia, the medial facet width was 12% of the lateral facet width, but in healthy knees, the mean was 57% [26].

**Table 3:** Comparison of Wiberg types of the patella R: right; L: left; N: sample size; n: number

Study (year)	Country	N	Type I n(%)	Type II n(%)	Type III n(%)	
Bisht et al. (2022) [5]	India	100 (50 R, 50 L)	11 (11)	85 (85)	4 (4)	
						Agarwal et al. (2018) [17]	India	60 (34R, 26 L)	-	52 (86.66)	8 (13.34)	
Maia et al. (2021) [20]	Brazil	59 (28 R, 31 L)	9 (15.2)	50 (84.8)	-							
Wiberg et al. (1941) [8]	Sweden	25	3 (12)	21 (84)	1 (4)	
Taj et al. (2022) [9]	India	50 (26 R, 24 L)	2 (4)	46 (92)	2 (4)	
Murugan et al. (2017) [21]	India	65	7 (10.8)	58 (89.2)	-	
This study	Turkey	168 (82 R, 86 L)	13 (7.73)	109 (64.88)	46 (27.38)	

The significance of Wiberg's classification is the knowledge that the position of the vertical joint ridge changes due to changes in the shape of the patella. In Wiberg Type 3, the vertical ridge has been displaced most medially, and the medial facet has turned into a convex structure. As a result, the risk of joint arthrosis and patellofemoral dislocation increases. This indicates that each type of patella has a unique center point, and it is very important in determining the location of the patellar component during TKA. Moreover, the difference in patellar incision sizes between sexes should be taken into account in the design of patellar components [27].

Li et al. assessed patellar morphology differences between patients with trochlear dysplasia and individuals with healthy knees. The WA was measured as follows: 137.2±5.7 degrees in male trochlear dysplasia patients 135.0±5.2 degrees in male individuals with healthy knees, and 130.3±6.8 degrees in female trochlear dysplasia patients compared to 129.2±4.5 degrees in females with healthy knees. The WA was found more in trochlear dysplasia patients than in healthy knees, but no significant difference was detected between these groups [28]. In contrast to Li et al., Fucentese et al. measured WA lower in trochlear dysplasia patients (125.64 degrees) than in healthy knees (129.36 degrees) [22]. In our study, WA was measured at 121.73±6.26 degrees, which is lower than Li et al. and Fucentese et al. [22,28].

The patella is the largest sesamoid bone, increasing the lever arm and allowing the quadriceps strength in the knee extensor apparatus to strengthen by 50% [29]. This largest sesamoid bone provides stability to the knee joint, protects it from its anterior aspect, and aids in transmitting the force of the quadriceps femoris contraction to the ligamentum patellae [1]. Also, the patella is a key bone in surgical procedures like TKA, medial patellofemoral ligament (MPFL) reconstruction, trochleoplasty, and many other surgical procedures [30-32]. When an implant is selected for patellar reconstruction, it results in losing the quadriceps lever and its effectiveness, limiting movement, and premature implant wear and tear, all of which worsen knee pain and instability. Finding the proper patellar implant size is essential for the success of patellofemoral or total knee replacements. Surgeons may be able to estimate the depth of resection in total knee replacement procedures by using their knowledge of patellar thickness [33].

Total knee arthroplasty stands out as the optimal surgical procedure; nevertheless, surgeons performing it face challenges related to patellofemoral joint derangement. Postoperative complications persist, with disability and patellar thickness emerging as crucial factors. A thinner patella may reduce friction but pose risks of stress fractures and instability. Increased PT might enhance quadriceps movement at a low knee flexion angle but limit the range of knee motions, potentially leading to patellar subluxation. Both thicker and thinner patellae have a smaller contact area compared to the normal size. Considering these factors, it is advisable to determine population, geography, sex, and age-specific patellar thickness during prosthesis implantation [34,35].

Iranpour et al. evaluated the width/thickness ratio of the patella to determine the optimal size for prosthesis implants in knee arthroplasty. The PRT was calculated as 2.1±0.28 [4]. In a study by Muhammed et al., they examined the PRT, which represents the ratio of thickness to width. This measurement can serve as a reference for estimating the premorbid patellar thickness. Their findings revealed a statistically significant difference in PRT between males (0.48±0.03) and females (0.45±0.04) (P<0.001). They also evaluated PFTR, which was calculated as 0.60±0.05 in males and 0.63±0.05 in females (P<0.001). [35]. We calculated the patellar width-to-thickness ratio (PRT) using a method similar to Iranpour et al. The calculated ratio was 2.10±0.15, which was consistent with Iranpour et al.'s calculation [4]. The PFTR was calculated at 0.56±0.06 in our study, which was in accordance with Muhammed et al. [35]. When it comes to patella reconstruction after arthroplasty, there are other variables to consider. The various designs of the femoral trochlea and patellar button, which essentially reproduce the natural morphological characteristics, will undoubtedly have an impact on the patella PRT [4,14,35]. We do not yet know if it is desirable or suitable to turn an aberrant patella into one with a normal PRT in cases of highly abnormal patellofemoral joints, such as those with primary patellofemoral arthritis related to trochlear dysplasia [4,35]. There is a real risk of overfilling the joint in these difficult cases, but this ratio gives the surgeon some numerical rules of thumb to start with. However, most patellae regrown during total condylar knee arthroplasty will not have significant morphological defects in the patellofemoral joint. In these cases, a PRT of 2.00 can be a starting point on which to base decisions about patellar reconstructive surgery [4, 32, 35].

Limitations

The limitation of the study is that the age and sex differences of the patella could not be revealed since the age and sex of the evaluated bones were unknown. Further research is needed for more details.

## Conclusions

In conclusion, our study demonstrated the anatomical structure of the patella through the defined Wiberg classification and PRT measurements. In conditions such as osteoarthritis where reconstruction of the patellar articular surface is necessary, or in cases of reshaping the patellar surface of the femur, as in trochleoplasty, it is crucial to insert the patellar component anatomically. To ensure this, the morphometric measurements of the patella, which are necessary for designing the implant and for the surgeon to accurately insert it at the most suitable location on the patella, have been elucidated in this study. We anticipate that this study will prove valuable to knee surgeons performing surgical procedures and to clinicians assessing conditions in the region. We hope that the data in this study will contribute to future studies.
